# Electron acceptor redox potential globally regulates transcriptomic profiling in *Shewanella decolorationis* S12

**DOI:** 10.1038/srep31143

**Published:** 2016-08-09

**Authors:** Yingli Lian, Yonggang Yang, Jun Guo, Yan Wang, Xiaojing Li, Yun Fang, Lixia Gan, Meiying Xu

**Affiliations:** 1 School of Bioscience and Bioengineering, South China University of Technology, Guangzhou 510006, China; 2 Guangdong Provincial Key Laboratory of Microbial Culture Collection and Application, Guangdong Institute of Microbiology, Guangzhou 510070, China; 3 State Key Laboratory of Applied Microbiology Southern China, Guangzhou 510070, China; 4 Science and Technology Library of Guangdong Province, Guangzhou 510070, China.

## Abstract

Electron acceptor redox potential (EARP) was presumed to be a determining factor for microbial metabolism in many natural and engineered processes. However, little is known about the potentially global effects of EARP on bacteria. In this study, we compared the physiological and transcriptomic properties of *Shewanella decolorationis* S12 respiring with different EARPs in microbial electrochemical systems to avoid the effects caused by the other physicochemical properties of real electron acceptor. Results showed that the metabolic activities of strain S12 were nonlinear responses to EARP. The tricarboxylic acid cycle for central carbon metabolism was down-regulated while glyoxylate shunt was up-regulated at 0.8 V compared to 0.2 and −0.2 V, which suggested that EARP is an important but not the only determinant for metabolic pathways of strain S12. Moreover, few cytochrome *c* genes were differentially expressed at different EARPs. The energy intensive flagella assembly and assimilatory sulfur metabolism pathways were significantly enriched at 0.8 V, which suggested strain S12 had stronger electrokinesis behavior and oxidative stress-response at high EARP. This study provides the first global information of EARP regulations on microbial metabolism, which will be helpful for understanding microorganism respiration.

Microbial respirations play important roles in various natural biogeochemical processes and engineered biosystems^1^. An extensively reported and interesting phenomenon in microbial respiration is that the substrate diversity and metabolic efficiency of microorganisms vary according to the electron acceptors^2^,^3^. For instance, *Shewanella* species can utilize diverse organic compounds as electron donors in respiration with oxygen, while the electron donors were limited to acetate and lactate in nitrate respiration and only lactate for iron and fumarate respiration^3^. It was further evidenced that *S. oneidensis* MR-1 performed a complete tricarboxylic acid cycle (TCA) in respiration with oxygen or trimethylamine N-oxide (TMAO), but a branched-TCA pathway in anaerobic fumarate respiration^4^,^5^. In addition to the central carbon metabolism, microbial electron transfer pathways may also switch according to different electron acceptors^6^,^7^.

Electron acceptor redox potential (EARP) was considered to be a key factor in determining microbial metabolism^6^. The EARP in microbial respiration generally ranged from −0.2 (CO_2_/CH_4_) to 0.8 V (O_2_/CO_2_)^7^. According to the Gibbs free energy calculation, the free energy available for microbial metabolism, in theory, should increase with EARP. For instance, every 0.3 V increase in EARP can release −58 kJ more energy (~ one ATP) per two electrons, which may enable microbes to overcome the energy gap in metabolizing many kinds of refractory compounds^6^. However, it is challenging to study the relationship between EARP and microbial metabolism activity because the naturally existing electron acceptors always contain different physicochemical properties (such as solubility, molecular composition), which may also affect the microbial metabolisms.

Microbial electrochemical systems (MES) have potential applications in many fields such as bioenergy, bioremediation, bioelectrosysnthesis, biosensor, as well as microbiology researches^8^. The solid electrode with accurately poised redox potential in MES provides an ideal tool to observe the regulation of EARP on microorganisms^9^,^10^. In recent years, the influence of anode potential on power generation, microbial community and contaminants degradation have been intensively studied for MES optimization. However, many studies showed the biofilm growth, current generation and substrate consumption were nonlinear responses to EARP, which is inconsistent with the thermodynamics theory^6^,^11^. Microbial respiration with electrode is a central driving force for the complex bioelectrochemical functions in MES, while only few studies paid attention on the regulation of EARP on the intracellular processes, or only focused on several EARP-specific enzymes involved in TCA cycle and the electron transfer process^10^,^12^. Based on previous reports^6^,^7^,^10^,^12^, we hypothesized that, in addition to genes encoding the components in TCA cycle and electron transfer chain, some important but ignored energy intensive metabolic pathways may be enriched at more positive potentials which may be responsible for the nonlinear responses. In order to verify our hypothesis, global and comprehensive information of EARP regulation on microorganism metabolism is essential.

*Shewanella* genus has both direct and indirect electron transfer pathways to electrodes and thus was used as one of the model electrode-respiring bacteria^13^,^14^,^15^. In this study, the physiological performances and transcriptome of *S.decolorationis* S12 respiring with electrodes with different redox potentials (−0.2, 0.2 and 0.8 V vs standard hydrogen potential, comparable to the EARP of natural common electron acceptors such as goethite (−0.18 V), sulfate (−0.21 V), S_3_O_6_^2−^ (0.22 V), O_2_ (0.81 V)) were comparatively analyzed^7^. This study provides new insights into our understanding of bacterial responses to EARP and offers important information on the regulation of bacterial metabolism.

## Results

### Physiological performances of *S. decolorationis* respiring with different EARPs

**Figure 1:**
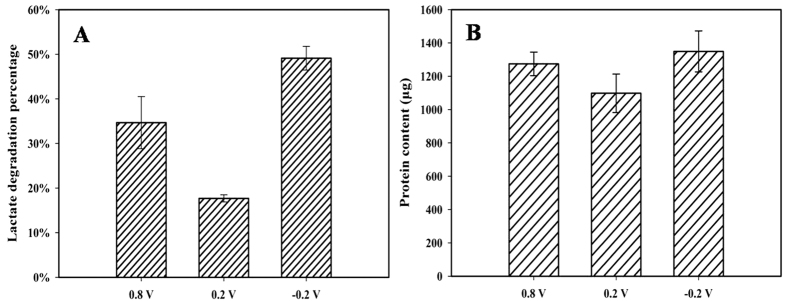
Lactate consumption (**A**) and biomass yield (**B**) under different EARPs.

*S.decolorationis* S12 showed the highest lactate oxidizing rate (49.1%) in MES with −0.2 V anode potential (MES_−0.2_), followed with that in MES with 0.8 V anode potential (MES_0.8_) (34.8%) and MES with 0.2 V anode potential (MES_0.2_) (17.7%) (Fig. 1A). Lactate oxidization capacity exhibited significantly difference (*p* = 0.004, ANOVA) in MES_−0.2_ and MES_0.2_. It was consistent with the protein-based biomass comparison (MES_−0.2_ > MES_0.8_ > MES_0.2_) (Fig. 1B), as lactate is the sole carbon resource for cellular component synthesis.

Confocal laser scanning microscopy (CLSM) results showed that only scarce cell clusters were observed on the anode surfaces in all MES which can be attributed to the short cultivation time (8 h, Supplementary Fig. S1). Anodes in MES_−0.2_ and MES_0.8_ showed denser cell cluster and larger biofilm-covered area than anode in MES_0.2_, which is in line with the total biomass quantification results (Fig. 1B). However, more unviable cells (with red fluorescence) were observed on the 0.8 V electrode than on the others, indicating an unfavorable biofilm growth status on the electrode surface in MES_0.8_.

As a key coenzyme, NADH participates in a number of metabolic reactions and the ratio of NAD^+^/NADH has been suggested to be a key factor in determining cell metabolism and can reflect the extracellular redox status^16^. However, in our study the NAD^+^/NADH ratios showed no significant difference among MES_−0.2_, MES_0.8_ and MES_0.2_ (Supplementary Fig. S2), which is in line with a recent study with *Geobacter sulfurreducens* that the NAD^+^/NADH ratio showed no difference under four potentials of 110, 10, −90, and −190 mV^17^.

### Global gene expression pattern

**Figure 2:**
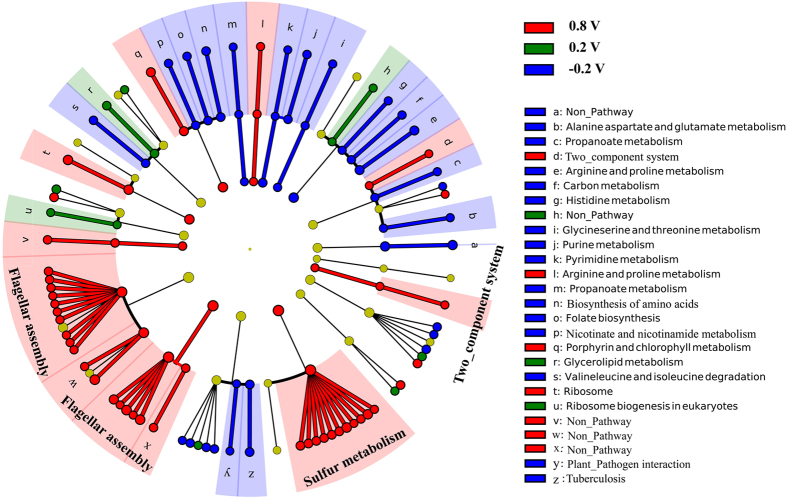
All significant regulated genes and enriched pathways under different EARPs. The innermost layer of circle is COG categories, the second layer of circle is metabolism pathways and the outermost layer of circle is genes. The flagellar assembly pathway and the sulfur metabolism pathway were especially enriched in MES_0.8_.

Sequencing resulted in a total number of ~10.8 to 15.2 million reads per sample and more than 93.8% of the reads could be mapped to the annotated strain S12 genome (accession number AXZL00000000). A total of 4215 unigenes (4219 unigenes of reference genome) had mapped reads and 50.91% (2255/4215) of the unigenes could be annotated in KEGG database. Mean FPKM value (Fragment per kb per million mapped reads) was used for calculating fold change. Differentially expressed unigenes were identified with FDR (False discovery rate) ≤0.05 and |log_2_fold change| ≥ 1. A total of 369 genes were differentially transcripted among different samples, of which 282 genes in log_2_(FPKM_0.8 V_/FPKM_0.2V_), 255 genes in log_2_(FPKM_0.8 V_/FPKM_−0.2V_) and 45 genes in log_2_(FPKM_0.2 V_/FPKM_−0.2V_). Less transcriptome differences were observed between MES_−0.2_ and MES_0.2_ relative to those with MES_0.8_, suggesting that strain S12 respiring with −0.2 and 0.2 V electrode performed more similar metabolic profile compared to cells with 0.8 V electrode. All those 369 differentially transcripted genes were divided into 20 categories according to COG classification (Fig. 2). Furthermore, metabolic pathway enrichment analysis exhibited flagellar assembly (corrected *P*-value = 0.001) and sulfur metabolism (corrected *P*-value = 0.001) were differently enriched in MES_0.8_ compared with MES_0.2_ and MES_−0.2_ (Fig. 2). Several noteworthy genes involved in central carbon metabolism and electron transfer process were highlighted in the enrichment analysis.

### Central carbon metabolism

**Figure 3:**
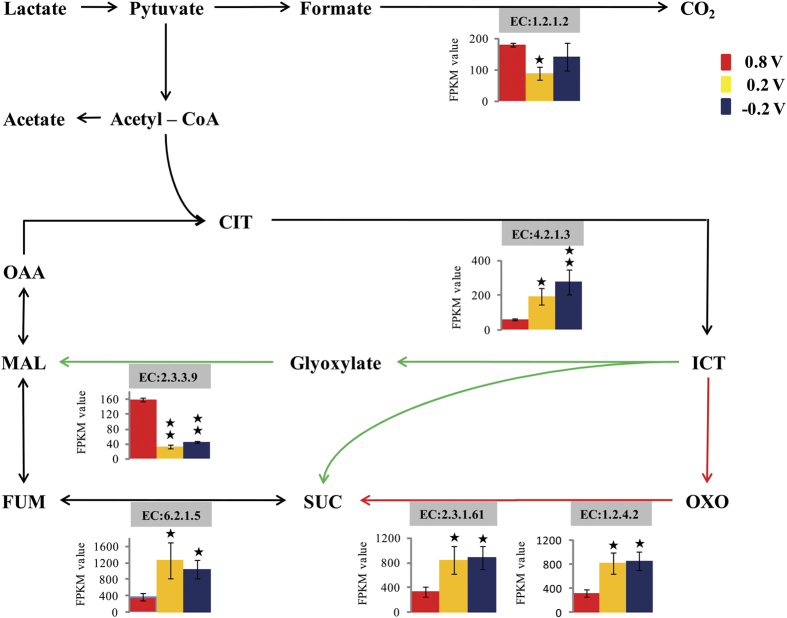
Central carbon metabolism of strain S12 at different EARPs. The red solid arrow lines denote TCA cycle key steps and the green ones denote key steps of glyoxylate pathway. The black ★ and longitudinal double ★ respectively denote the expression is significant (FDR ≤ 0.05 and |log_2_FC| ≥ 1) and extremely significant (FDR ≤ 0.01 and |log_2_FC| ≥ 2) differences with comparing to 0.8 V. Name of enzyme: EC:1.2.1.2, formate dehydrogenase; EC:4.2.1.3, aconitatehydratase; EC:2.3.3.9, malate synthase; EC:6.2.1.5, succinyl-CoA synthetase; EC: 2.3.1.61, 2-oxoglutarate dehydrogenase E2 component; EC: 1.2.4.2, 2, 2-oxoglutarate dehydrogenase E1 component. Abbreviations: CIT, citrate; ICT, isocitrate; OXO, 2-ketoglutarate; SUC, succinate; FUM, fumarate; MAL, malate; OAA, oxaloacetate.

Carbon metabolism is one of the most important pathways determining the substrate uptake, energy generation and cell growth, in which TCA cycle plays a central role^12^. In this study, several genes encoding key enzymes in TCA pathway were significantly up-regulated in MES_0.2_ and MES_−0.2_ compared with those in MES_0.8_ (Fig. 3), including aconitate hydratase (converting citrate to isocitrate), 2-oxoglutarate dehydrogenase E2 component and 2-oxoglutarate dehydrogenase E1 component (converting 2-oxoglutarate to succinate), succinyl-CoA synthetase (catalyzing the redox reaction between fumarate and succinate). Moreover, 2-methylcitratesynthase gene (*prp C*) which contributes to the anaerobic acetyl-CoA metabolism, the gate in TCA cycle, also showed significantly higher transcription level under the two lower EARPs. These results indicated that the TCA pathway of *S. decolorationis* S12 was enriched in MES_0.2_ and MES_−0.2_ compared with MES_0.8_. Remarkably, formate dehydrogenase and the key enzyme of malate synthase in glyoxylate shunt were significantly up-regulated in MES_0.8_ relative to MES_0.2_ and MES_−0.2_ (Fig. 3).

### Respiration and energy generation

The capacity that bacteria transfer electrons to extracellular acceptors (e.g. mineral oxides, humics and electrodes) is fundamentally important for various biogeochemical processes and the development of MES. Therefore, it is one of the most interesting issue to investigate whether and how the bacteria regulate the electron transfer pathway in response to EARP. By analyzing 53 differentially transcripted genes attributed to electron transfer and energy generation processes, the transcription pattern of those genes under −0.2 and 0.2 V were similar and significantly distinguished from that under 0.8 V (Supplementary Fig. S3). Among those, 6 genes encoding different types of cytochromes were significantly up-regulated at 0.8 V while 3 were up-regulated at 0.2 or −0.2 V (Table 1), suggesting that some electron transfer components in *S. decolorationis* S12 are EARP-specific. The outer membrane cytochrome *c* (e.g. MtrC, OmcA) and electron mediator flavins have been proven to be essential for *Shewanella* extracellular electron transfer, however no significant difference was detected for these redox components^13^.

### Flagellar assembly and sulfur metabolism pathways

Flagellum is a key cellular appendage for bacteria motility, movement toward solid surfaces and bacterial electrode respiration^18^. The energy intensive flagella synthesis and functioning are tightly related to cell energy conservation and consumption^19^. Our results showed that flagellar assembly pathway was significantly enriched (corrected *P*-value = 0.001) in MES_0.8_ (Table 2). Moreover, genes encoding two-component system components (MCP, FliC, CheV and CheR), for regulating flagellum switch, were also upregulated at 0.8 V. It has been reported that *Shewanella* biofilm cells showed depressed flagellum assemble compared with planktonic cells as flagella were no more needed after cells were embeded in biofilms^20^.

**Figure 4:**
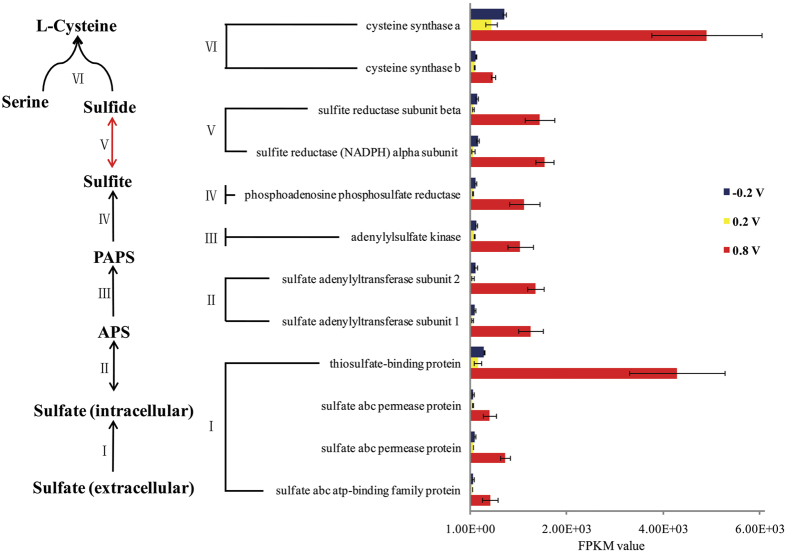
Significantly enriched sulfur metabolism pathway in MES_0.8_. The roman numerals beside arrows indicate steps catalyzed by the corresponding enzymes in the right.

Assimilatory sulfur metabolism was one of the most significantly enriched pathways in MES_0.8_ (corrected *P*-value = 0.001) (Fig. 4). Twelve genes involved in this pathway were significantly up-regulated which highlighted a completely activated sulfur assimilation pathway including extracellular sulfate transport, sulfite and sulfide generation and cysteine synthesis. Especially, the sulfite reductase (NADPH) alpha subunit, which is the key gene in sulfur metabolism pathway, was significantly upregulated in MES_0.8_ (23 and 9.5 fold higher than that in MES_0.2_ and MES-_0.2_, respectively)^21^. It was suggested that strain S12 converted lots of energy and electrons or performed cysteine synthesis in MES_0.8_. Cysteine is an essential amino acid for many oxidoreductases (e.g. cytochromes, thioredoxins and Fe-S proteins). The enrichment of cysteine synthesis pathway was consistent with the results that more genes encoding cytochromes or some other cysteine-containing enzymes involved in energy generation were upregulated under 0.8 V. Moreover, cysteine itself can function as electron mediator in bacterial electron transfer^22^.

## Discussion

Bacterial respiration is a central driving force in various environmental and engineering processes. The EARP has been considered to play a key role in determining the bacteria energy conservation, cell growth and activities, and become one of the key factors in understanding biogeochemical processes and optimizing microbial reactors. In this study, a significant partial of genes involved in central carbon metabolism, sulfur metabolism, cell motility and stress-response showed specific responses to EARP by analyzing the global transcriptomic profiling of *S. decolorationis* S12.

On the central carbon metabolism, our results suggested a higher TCA cycle activity with lower EARP. Consistently, Matsuda *et al*. reported that the TCA pathway of *S. oneidensis* MR-1can be activated by decreasing the EARP from 0.4 to 0 V or lower, under which the expressions of rate-limiting enzymes in TCA pathway were up-regulated^10^. However, Gobbler *et al*. suggested a higher-activated TCA cycle of *S. oneidensis* MR-1 and more biofilm cells under higher EARP^12^. Regarding the discrepancy, it should be noted that our transcriptome results mainly come from the planktonic cells rather than biofilms, because planktonic cells play a dominant role in both electricity generation (via secreting electron shuttles) and biomass yield in *Shewanella* MES^15^,^23^. Previous work reported the protein expression profile of *Shewanella* biofilm cells was significantly different with planktonic cells^20^. It is possible that the central carbon metabolism between biofilm and planktonic cells of *Shewanella* has different responses to EARP. Unlike the other model electrode respiring bacteria genus *Geobacter*, *Shewanella* species have anapleurotic reactions through glyoxylate shunt which can reduce the oxidation of carbon substrate by TCA cycle and save carbon sources for biosynthesis^24^,^25^. The up-regulated glyoxylate shunt indicated an incomplete TCA cycle under higher EARP (0.8 V vs 0.2 V and −0.2 V). Therefore, it seems that the central carbon metabolism of strain S12 switched from complete TCA cycle to glyoxylate shunt along with the increase EARP. Moreover, it was reported that formate oxidation has a contribution on the growth rate and yield of *Shewanella oneidensis* strain MR-1 under anaerobic conditions^26^. The formate dehydrogenase being upregulated significantly in MES_0.8_ could be responsible for the higher lactate degradation and growth rate.

It has been reported that *Shewanella* species including *S. decolorationis* S12 can utilize lactate as carbon resource and electron donor in oxygen respiration though complete TCA cycle but low active or incomplete TCA cycle under anaerobic conditions^4^,^5^. Although the EARP of MES_0.8_ is close to oxygen (0.81 V vs SHE), strain S12 in MES_0.8_ performed an up-regulated glyoxylate shunt and incomplete TCA cycle pathway. It can be seen that EARP has significant impact on microbial metabolism pathways, but it is not the only determining factor. The other factors of an electron acceptor such as the solubility, molecular structure may also play essential roles in regulating bacterial metabolism. It should be noted that, different with the natural electron acceptors, the inherent electrochemical processes such as electrode kinetics, the overpotential of electroactive materials and extremely large electrostatic interaction in the electric double layer in MES may also have interactions with bacteria cells which should be considered in further studies.

The different transcriptomic profile of several cytochromes which participated in electron transfer supported the previously reported electrochemical phenomena, that electrode respiring bacteria showed different voltammetry behaviors on electrodes with different redox potentials which indicated different redox-species were generated on the electrode surface^6^,^7^. A recent report showed an ImcH/CbcL system in *Geobacter* for sensing the EARP switch below or above 0.1 V^6^, which also indicated possible different redox sensing strategy was used between *Shewanella* and *Geobacter*. Despite the transcription levels of *S. decolorationis* S12 outer membrane cytochrome *c* showed no difference here, the conformation flexibility of the outer membrane cytochrome *c* can facilitate *Shewanella* to adapt to transfer electrons to electron acceptors at different EARPs. Therefore, the self-regulation of *Shewanella* to electron acceptors may be a complex processes including inter- and intra- molecular changes.

It is interesting to note that the higher EARP did not resulted proportional biomass increase in MES_0.8_ compared with MES_0.2_ and MES_−0.2_ which can be explained by the high oxidative stress and energy-inefficiency redundancy pathways (e.g. flagellar assembly and assimilatory sulfur metabolism)^19^,^27^. Planktonic cells play a dominant role in electrode respiration in *Shewanella* MESs, especially before a mass of biofilms generated on electrode surface^23^,^28^. Electrokinesis (i.e. bacteria rapid swim around solid electron acceptor) has been demonstrated to be a crucial process in the electron transfer of planktonic cells to electrodes. Higher electrode potential could stimulate *Shewanella* electrokinesis near the electrode surface by which the planktonic cells could transfer electrons to electrode at high frequency via a “touch and go” model^28^. The higher transcription level of flagella coding genes at high EARP is in line with the electrokinesis theory in *Shewanella* extracellular respiration and consistent with the less biofilm formation on the 0.8 V electrode surface (Supplementary Fig. S1). However, it remains mystery that why *S. decolorationis* cells preferred to swim rather than be sessile on electrode surface at 0.8 V, even though flagella synthesis and cell movement is highly energy-cost and no electron transporting moiety is included in those appendages^14^.

Furthermore, high EARP may cause oxidative stress to bacterial cells by generating reactive oxygen species (ROS)^29^, which may explain that more biofilm cells with impaired membrane (with red fluorescence) can be founded on the 0.8 V electrode. Synthesis of glutathione or other low molecular weight thiols is a key strategy for bacteria addressing oxidative stress^30^. Cysteine is an essential precursor for generating or endowing those compounds with ability to remove ROS. It has been reported that cysteine synthesis of *Shewanella* or some other bacteria were increased under H_2_O_2_ or alkaline stress^31^. Therefore, it was presumed that the enrichment of assimilatory sulfur metabolism may be a strategy of *Shewanella* to address the oxidative stress caused by the high electrode potential.

All results in this study indicated that EARP, a key but not the only determinant for central carbon metabolism, performed global regulation on the transcriptomic profile of strain S12. Briefly, *S. decolorationis* S12 showed an EARP-specific central carbon metabolism and electron transfer pathway. The energy intensive flagella assembly and assimilatory sulfur metabolism pathway were significantly enriched under higher EARP as strategies for electrokinesis and anti-oxidative stress, respectively. The results not only extend our understanding on microbial respiration and metabolism processes but also provide implications for the developments of MES and some other microbial technologies.

## Materials and Methods

### Bacterial strains and MESs operation

*S. decolorationis* strain S12 was isolated from activated sludge and preserved in our laboratory^32^. The strain can respire with various electron acceptors, such as Fe (III), fumarate, nitrate, azo dyes and electrode^33^,^34^,^35^. A single colony of strain S12 was cultured aerobically in 100 ml LB at 30 °C overnight. The cells were centrifuged and washed three times with lactate medium (LM, 10 mM, pH7.5) to be used as inoculums for MES.

Dual-chamber with potentiostat (CHI 1040C) MES were assembled and sterilized as previously reported^15^. Briefly, the anode chamber of MES contained a graphite anode, a reference electrode (AgCl/Ag, 0.195 V vs standard hydrogen electrode (SHE)) and 100 mL of lactate medium (LM). The cathode chamber contained a graphite cathode and 100 mL of PBS with 50 mM potassium ferricyanide. Anode potentials in different MESs were poised at −0.2 V, 0.2 V and 0.8 V. All samples had three repetitions and all mentioned potentials had been converted to vs SHE. Initial bacterial density in anode culture was 0.05 (OD_600_, optical density at a wavelength of 600 nm).

### Chemical and physiological analysis

Lactate concentration in culture medium and protein-based biomass volume of the planktonic cells were by HPLC and a protein quantification assay, as previously reported^36^. SigmaPlot 11.0 was used for data (mean and standard deviation) statistical analysis. CLSM was used to analyze the development and structure of the biofilms attached on anode surfaces^15^. Before CLSM observation, biofilm samples were stained with LIVE/DEAD BacLight staining kit (Molecular Probes) which can distinguished viable (with green fluorescence) or unviable or stressed (with red fluorescence) bacterial cells. Randomly sampled view fields were observed and analyzed for each anode biofilm. To obtain three-dimensional structure information, the biofilm sample was observed under the “z-Stack” model of the Zen software (Zeiss)^15^.

### RNA extraction and transcriptome analysis

For transcriptome analysis, *S. decolorationis* S12 cells cultured in LM with different EARPs were collected for RNA extraction as described by Holmes *et al*.^37^. Planktonic cells were included in transcriptome analysis due to their significant role in electricity generation with electron mediator^23^,^38^. RNA protect was immediately added to the cell deposits and then stored at −80 °C before RNA extraction. RNA was extracted using the commercial RNeasy Mini Kit (Qiagen, Germany) with DNase (RNase-free, Takara) treatment according to the manufacturer’s instruction. The extracted RNA was quantified and evaluated by Agilent 2100 bioanalyzer and Caliper Labchip GX. Amplified fragments were sequenced using Illumina HiSeq™ 2500.

Raw sequence data were filtered to remove those containing adapter and reads with more than 10% unknown nucleotides, and reads with more than 50% of low quality base (value ≤5). Clean reads were mapped into the transcriptome reference database using Tophat 2.0.1 and Samtools 0.1.18.0. Less than 3 mismatch bases were permitted, and unique mapped reads were obtained. Cufflinks 2.0.0 software was used for calculating the FPKM value and difference analysis. P-value was used to evaluate the difference of gene transcription and FDR was used to determine the threshold of P-value^39^.

Genes were annotated in COG (Cluster of Orthologous Groups of protein) database for identification of orthologous proteins and KEGG (Kyoto Encyclopedia of Genes and Genomes) database for annotation and metabolic pathway analysis. Enrichment metabolic pathways were analyzed by both KOBAS 2.0 and Galaxy LEfSe, which set *P*-value < 0.05 as statistically significant.

## Additional Information

**How to cite this article**: Lian, Y. *et al*. Electron acceptor redox potential globally regulates transcriptomic profiling in *Shewanella decolorationis* S12. *Sci. Rep*. **6**, 31143; doi: 10.1038/srep31143 (2016).

## Supplementary Material

Supplementary Information

## Figures and Tables

**Table 1:** The significant differentially expressed cytochrome-associated genes.

COG number: Gene products	Average FPKM			|Log_2_ fold change| ≥ 1 and FDR value ≤ 0.001)		
	0.8 V	0.2 V	−0.2 V	0.8 V/0.2 V	0.8 V/−0.2 V	0.2 V/−0.2 V
COG2993:cbb3-type cytochrome c oxidase	649.99	1017.05	1391.18	—	−1.10	—
COG0843:cytochrome o ubiquinol oxidase	104.14	526.40	362.22	−2.34	−1.80	—
COG1845:cytochrome o ubiquinol oxidase	87.57	442.74	318.73	−2.34	−1.86	—
COG1271:cytochrome bd ubiquinol oxidase	191.67	80.93	63.48	1.24	1.59	—
COG1294:cytochrome d ubiquinol subunit	345.82	133.25	240.51	1.38	—	—
^a^cytochrome b561	126.39	71.17	53.78	—	1.23	—
^a^cytochrome c3	1583.96	739.39	1332.91	1.10	—	—
COG1969:quinone-reactive Ni/Fe-hydrogenase cytochrome b	320.66	115.84	170.20	1.47	—	—
COG3005:NapC/NirT cytochrome c	969.56	436.51	541.94	1.15	—	—

“—” denotes no significant difference; “a” denotes no available COG number.

**Table 2:** The differentially expressed flagellar genes.

COG number: Gene products	Average FPKM			|Log_2_ fold change| ≥ 1 and FDR value ≤ 0.001)		
	0.8 V	0.2 V	−0.2 V	0.8 V/0.2 V	0.8 V/−0.2 V	0.2 V/−0.2 V
COG4787:flagellar basal body rod protein	699.76	330.73	137.53	1.08	2.35	1.27
COG1815:flagellar basal-body rod protein	1886.81	732.51	625.15	1.37	1.59	—
COG1558:flagellar basal body rod protein	827.92	321.44	265.88	1.36	1.64	—
COG4786:flagellar basal-body rod protein	740.53	343.42	172.64	1.11	2.10	—
COG1677:flagellar hook-basal body complex	464.24	166.95	107.72	1.48	2.11	—
COG3144:flagellar hook-length control protein	563.20	250.10	200.60	1.17	1.49	—
COG2063:flagellar l-ring protein	473.54	225.78	117.25	1.07	2.01	—
COG1705:flagellar rod assembly protein muramidase	849.26	362.41	243.21	1.23	1.80	—
COG1334:flagellar protein	5327.98	2001.53	2373.24	1.41	1.17	—
COG1516:flagellar protein	4673.94	1762.51	2155.63	1.41	1.12	—
COG1344:flagellin domain protein	4236.97	1575.13	1558.14	1.43	1.44	—
COG1298:flagellar biosynthesis protein	373.41	178.91	129.69	1.06	1.53	—
COG5616:outer membrane lipoprotein	1132.96	378.31	422.85	1.58	1.42	—
COG1344:flagellin	10069.50	3105.86	4591.29	1.70	—	—
COG1345:flagellar hook-associated 2 domain-containing protein	5127.99	1940.79	2448.05	1.40	—	—
COG1256:flagellar hook-associated protein	534.98	340.74	224.86	—	1.25	—
COG1344:flagellar hook-associated protein	605.44	362.38	271.36	—	1.16	—
COG1843:flagellar basal body rod modification protein	953.26	601.39	351.05	—	1.44	—
COG1706:flagellar p-ring protein	459.79	245.52	123.78	—	1.89	—
^a^flagella biosynthesis chaperone	3614.49	1325.25	1606.80	1.45	1.17	—
^a^flagella assembly protein	428.83	265.91	193.69	—	1.15	—

“—” denotes no significant difference; “a” denotes no available COG number.
