# Supplementary material for: Electron acceptor redox potential globally regulates transcriptomic profiling in Shewanella decolorationis S12
Source: Sci Rep. 2016 Aug 9;6:31143. doi: 10.1038/srep31143 (PMC4977559; doi:10.1038/srep31143)
Supplement: Supplementary Information [file srep31143-s1.docx]

**Supplementary Information**

**Electron acceptor redox potential globally regulates transcriptomic profiling in *Shewanella decolorationis* S12**

Yingli Lian^1, 2, 3^, Yonggang Yang^2, 3*^, Jun Guo^2, 3^, Yan Wang^4^, Xiaojing Li^2, 3^, Yun Fang^2, 3^, Lixia Gan^2, 3^, Meiying Xu^2, 3*^

^1^School of Bioscience and Bioengineering, South China University of Technology, Guangzhou 510006, China

^2^Guangdong Provincial Key Laboratory of Microbial Culture Collection and Application, Guangdong Institute of Microbiology, Guangzhou510070, China

^3^State Key Laboratory of Applied Microbiology Southern China, Guangzhou510070, China

^4^Science and Technology Library of Guangdong Province

* Corresponding author:

Meiying Xu, Guangdong Institute of Microbiology, 100# Central Xianlie road, Guangzhou, China 510070, Tel: +86 20 87684471, E-mail: [xumy@gdim.cn](mailto:xumy@gdim.cn).

Yonggang Yang, Guangdong Institute of Microbiology, 100# Central Xianlie road, Guangzhou, China 510070, Tel: +86 20 87684471, E-mail: yyg117@163.com.

**Supplementary Figures**


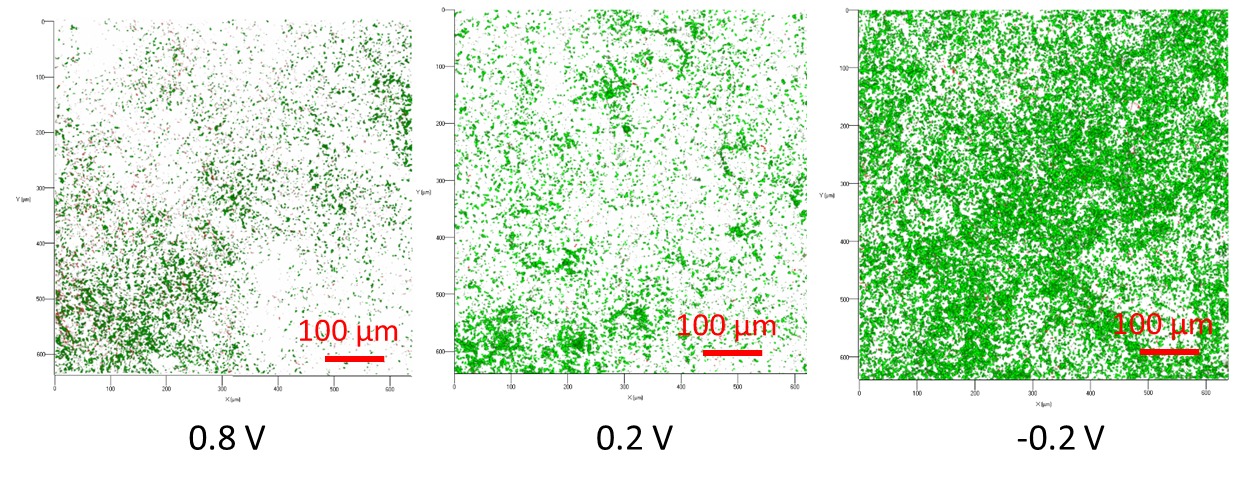


**Supplementary Figure S1. The 3D biofilm morphologies under different anode potentials (0.8 V, 0.2 V, -0.2 V).**

Cells stained green have integrated cell membrane and the red cells have damaged cell membrane.


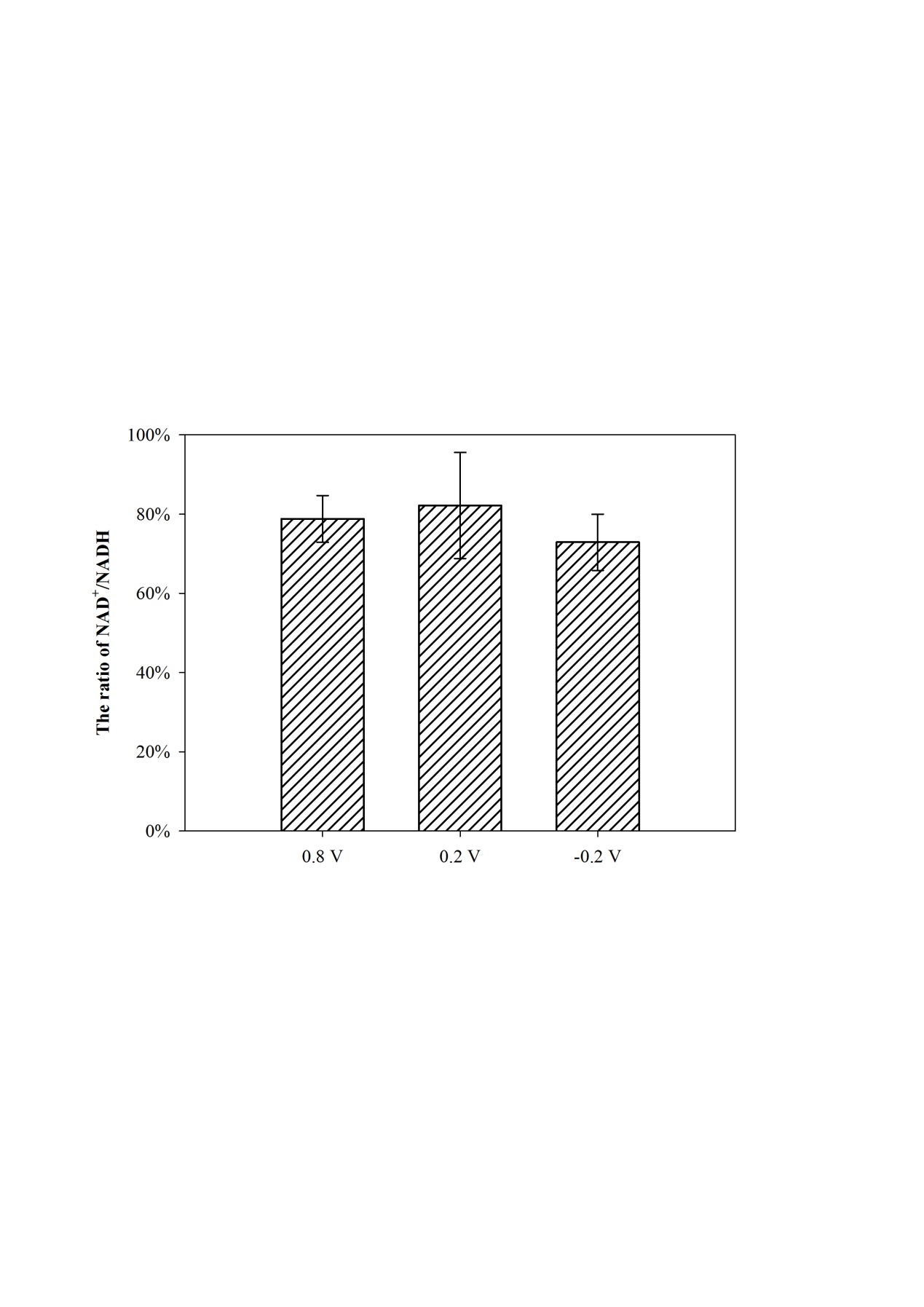


**Supplementary Figure S2. The cellular NAD^+^/NADH ratio.**


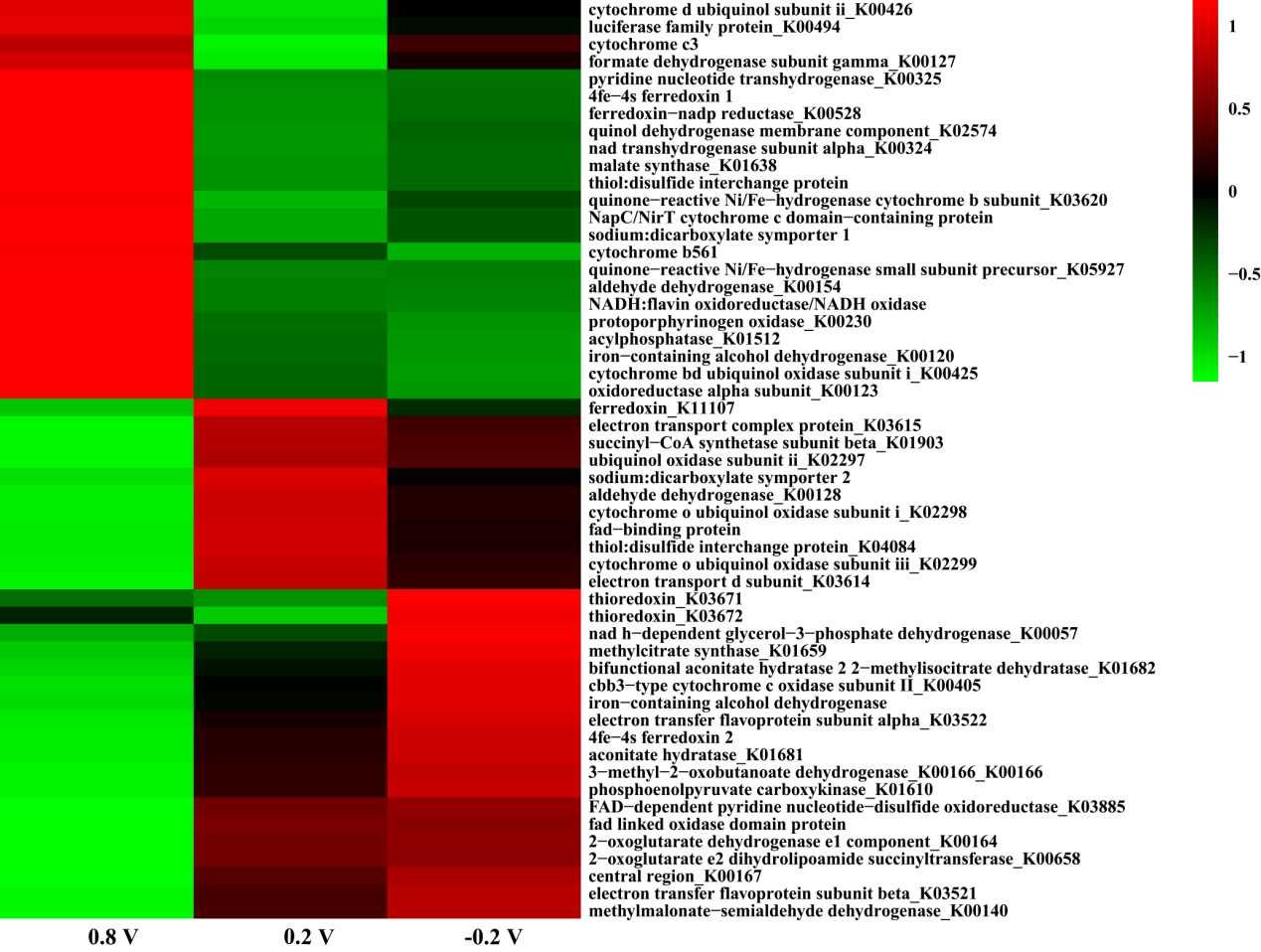


**Supplementary Figure S3. Heat map diagram of differentially expressed genes of electron transfer chain and energy production.**

Data for gene expression levels were normalized with z-score.
